# Deciphering the Role of Polyphenols in Sports Performance: From Nutritional Genomics to the Gut Microbiota toward Phytonutritional Epigenomics

**DOI:** 10.3390/nu12051265

**Published:** 2020-04-29

**Authors:** Vincenzo Sorrenti, Stefano Fortinguerra, Giada Caudullo, Alessandro Buriani

**Affiliations:** 1 Department of Pharmaceutical & Pharmacological Sciences, University of Padova, 35131 Padova, Italy; 2 Bendessere™ Study Center, Solgar Italia Multinutrient S.p.A., 35131 Padova, Italy; stefano.fortinguerra@gmail.com (S.F.); giada.caudullo@solgar.it (G.C.); alessandro.buriani@gmail.com (A.B.); 3 Maria Paola Belloni Center for Personalized Medicine, Data Medica Group (Synlab Limited), 35100 Padova, Italy

**Keywords:** phytonutrients, sports nutrition, epigenome, personalized medicine, microbiota, polyphenols, athletic performance, phytonutritional epigenomics

## Abstract

The individual response to nutrients and non-nutrient molecules can be largely affected by three important biological layers. The gut microbiome can alter the bioavailability of nutrients and other substances, the genome can influence molecule kinetics and dynamics, while the epigenome can modulate or amplify the properties of the genome. Today the use of omic techniques and bioinformatics, allow the construction of individual multilayer networks and thus the identification of personalized strategies that have recently been considered in all medical fields, including sports medicine. The composition of each athlete’s microbiome influences sports performance both directly by acting on energy metabolism and indirectly through the modulation of nutrient or non-nutrient molecule availability that ultimately affects the individual epigenome and the genome. Among non-nutrient molecules polyphenols can potentiate physical performances through different epigenetic mechanisms. Polyphenols interact with the gut microbiota, undergoing extensive metabolism to produce bioactive molecules, which act on transcription factors involved in mitochondrial biogenesis, antioxidant systems, glucose and lipid homeostasis, and DNA repair. This review focuses on polyphenols effects in sports performance considering the individual microbiota, epigenomic asset, and the genomic characteristics of athletes to understand how their supplementation could potentially help to modulate muscle inflammation and improve recovery.

## 1. Introduction

Certain individuals possess higher athletic abilities than others, although the mechanisms underlying these differences have only recently been better understood [1,2]. The interest in a personalized approach is increasing in sport to maximize each individual’s athletic ability both in endurance and strength sports [3,4]. Research has increasingly been focused on the genetic aspects that characterize elite athletes [5] as well as on their precise nutrition [3], and recently great interest has been directed towards plants and their phytocomplexes, which provides precious molecules of interest for sports performance [4]. Among the various phytochemicals, polyphenols represent a heterogeneous class of compounds with marked antioxidant and anti-inflammatory properties [6]. Polyphenols can act as key signal molecules when introduced into an organism. This can be achieved through a plethora of mechanisms, both direct on receptor proteins and indirect through the modulation of transcription factors or critical enzymes in survival and bioenergetic signal pathways [7,8,9]. One of the biggest challenges today in this field is understanding the interrelation mechanisms between polyphenols and the human body, also considering the fundamental role played by the intestinal microbiota in their absorption and bioavailability [10]. The current limit of polyphenol research in sports is the reductionist approach that usually drives the focus on specific outcomes in defined athletes, often in studies using small subject numbers, not considering the enormous potential these compounds might have on health if viewed in a systemic manner. 

The aim of this review is to contribute to better understand the effect of dietary polyphenols in sports performance, considering the influence of the individual genetic asset and the modulatory impact of the gut microbiota in polyphenol bioavailability and activity. In particular, nutritional and nutraceutical advice will be addressed in a practical way considering the possibility, nowadays, of genetic and microbiota analyzes that can support the personalization of a specific nutritional and nutraceutical regime for each athlete. A future perspective for the use of polyphenols in sports performance is also analyzed within the framework of a holistic approach, considering all the relevant biological layers, so that polyphenol effects on exercise and sports can be viewed taking into consideration both epigenetic and genetic aspects, as well as the impact of the gut microbiota. 

## 2. Nutritional Genomics and Sports Performance

Omic disciplines, including epigenomics (the study of the complete set of epigenetic modifications on the genetic material of a cell, known as the epigenome), aim at the complete characterization and quantification of pools of biological molecules that affect the structure, function, and dynamics of an organism. In nutrition, omics technologies are useful to customize food strategies for each individual, providing personalized dietary approaches [11,12].

The standardized nutritional approach—preferably related to guidelines for healthy nutrition such as those established by World Health Organization (WHO), rather than the recommended daily dosages in terms of daily calories, micronutrients, and macronutrients—should be revised and updated today considering the influence that genetic, environmental and microbiota factors have on each individual, to optimize nutritional and nutraceutical choices and promote the health of individuals according to their characteristics [13].

At the genetic level, two nutritional fields analyze the intricate relationships between nutrients, genes, and biological systems: nutrigenetics and nutrigenomics. Nutrigenetics aims to understand how our genetic background can modulate nutrients absorption, distribution, metabolism, and elimination (ADME), affecting response to diet. Nutrigenomics focuses on the individual sensitivity to nutrients in terms of influence on gene and protein expression and, subsequently, metabolite production, thus providing actionable information on the effects of diets and allowing effective personalized dietary-intervention strategies to prevent diet-related diseases [14,15].

One of the most useful applications of nutritional genomics is certainly in sports performance.

Genetic factors account for about 50% to 80% of interindividual variation in body mass, and this has an essential impact on muscular growth response [16]. Moreover, endocrine functions, muscle fibers composition, psychological aspects, and nutrition can have genotype-associated differences and influence athletic performance [17].

In particular, influences between genes and nutrients can affect nutrient availability and, subsequently, sport-related bodily functions. The amount and type of macronutrients (carbohydrates, lipids, and proteins) in a personalized nutritional regime is crucial for muscle functions and sports performance. In recent years significant progress has been made in describing how genetic variability can influence macronutrient absorption and functions. Numerous polymorphisms affecting essential genes involved in protein synthesis (L-amino acid transporter (LAT)1/2, mammalian target of rapamycin(mTOR), insulin-like growth factor(IGF)1), body weight (angiotensin-converting enzyme(ACE), fat mass and obesity-associated(FTO), insulin receptor substrate(IRS)2), carbohydrate absorption (glucose transporter(GLUT)4, thioredoxin interacting protein(TXNIP), monocarboxylate transporter(MCT)1/4) and lipid metabolism (lipin (LPIN)1, peroxisome proliferator activated receptor alpha (PPARA), fatty acid desaturase (FADS)1/2, apolipoprotein(APO)E) have been highlighted [3]. Amount and type of proteins, for example, are crucial for muscle growth and sports performance [18]. Protein intake and amino acid absorption and metabolism are different between individuals and are related first to protein quality, quantity and timing, and second to interindividual genetic background [4]. Genetic differences can affect the amounts of bioactive peptides derived from protein sources and, consequently, their use for muscle activity and growth [19]. Leucine, for example, is a critical factor for protein synthesis and enhances the activity of relevant kinases regulating translation processes such as the mTOR signaling pathway [20]. Genetic polymorphism in LAT1 and LAT2 gene, which encodes for branched-chain-amino-acid (BCAA) transporters, could influence the leucine absorption rate after ingestion contributing to reduce the amount of leucine available for protein synthesis [21]. Further, mTOR polymorphisms can have an impact on muscle growth and athlete performance in terms of nutrients absorption and protein synthesis [20,22]. Based on these genetic evidences, personalized nutritional strategies concerning protein intake in athletic individuals can be performed. More in-depth information has been published previously by our team [4]. For example, polymorphisms affecting tumor necrosis factor(TNF), MCT1, superoxide dismutase(SOD)2 genes have been linked to increased muscle fatigue and longer recovery time [23,24,25,26]. Athletes carrying these mutations should sustain physical activity for shorter durations with sufficient breaks between sets and longer recovery periods. They also have to daily introduce food rich in manganese (seafood, hazelnuts, whole wheat bread), hydroxymethyl butyrate (grapefruit, avocado), and ascorbic acid (orange, kiwi, black currant). Antioxidant and anti-inflammatory supplements such as polyphenols (curcumin, resveratrol, quercetin), omega-3, and glucosamine are also often recommended.

**Figure 1:**
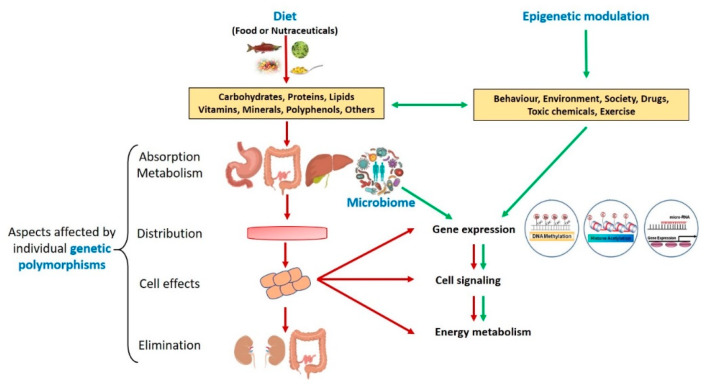
A new holistic view of nutrition with a focus on phytonutritional epigenomics: Influence of genetic mutations, epigenetic modulation, and the gut microbiome (for figure description, see the text).

Despite genetic testing for predicting sports performance and talent identification being continuously on the rise in the market, nutrigenetic and nutrigenomic analysis are often wrongly applied. This is primarily due to the complexity of interpreting the functional influence of each single polymorphism in nutrition, which can directly or indirectly affect different other genes, proteins, or metabolic pathways. Moreover, to increase complexity, nutritional genomics alone cannot explain all the metabolic phenomena occurring in the human body, and it should be proficiently matched with the study of the influences the microbiome has on human nutrition (Figure 1) [15].

### Diet, Microbiota, and Sports Performance

The intestinal microbiota is a thriving ecosystem of microorganisms composed of an estimated 100 trillion bacteria, viruses, fungi, and protozoa live in perfect symbiosis with our organism [27]. About 90% of the bacteria living in the human gastrointestinal tract belongs to 5 main *phyla*: Bacteroidetes characterized by some well-known genera such as *Prevotella* and *Bacteroides* [28]; Firmicutes to which the genera *Ruminococcus*, *Lactobacillus* and *Streptococcus* belong [28]; Actinobacteria, to which the genus *Bifidobacterium* belongs [29]; Proteobacteria—all Gram-negative and possibly pathogenic—and Verrucomicrobia, known mainly for the genus *Akkermansia* [27,30,31,32].

Microbiota diversity is crucial for our health. The greater the microbial diversity, the better the health of the individual. Reduced microbiota diversity of Western populations compared to less industrialized societies is mainly linked to the lack of microbiota accessible carbohydrates (MACs) essential to maintaining a thriving microbial ecosystem. MACs such as resistant starch, inulin, lignin, pectin, cellulose and fructooligosaccharides (FOS) are mainly derived from plant sources, but can also be of animal, fungal and algal origin [33,34].

Once reached the large intestine in their undigested form MACs become substrates for colon microbial fermentation, producing small molecules such as butyrate, acetate, and propionate known as short-chain fatty acids. SCFAs intersect with the host biochemistry through complex metabolic networks promoting anti-inflammatory, antioxidants, and immune-modulating mechanisms [11,35].

Physical activity has proven to modulate SCFA synthesis, exerting a particular influence on butyrate production [36]. In particular, in vivo data have shown that physical activity can increase butyrate synthesis by a relative increase in SCFA-producing bacteria [37,38].

Few human studies are available on the subject. In one of these, a group of rugby players was compared with sedentary controls. Questionnaires on the physical activity and diet were collected, and fecal samples were analyzed. Results showed higher microbial diversity in rugby players [38].

A recent study analyzed the microbiota of marathon runners and identified the critical presence of the bacteria *Veillonella atypica*. The inoculation of this bacteria into healthy mice significantly increased mice’s performance on the treadmill. This initial study highlights that *V. atypica* can be associated with athletic performance through the metabolic conversion of lactate in propionate [39].

Future studies are needed to understand the numerous bi-directional influences between physical activity, nutrition, and the gut microbiota.

## 3. Polyphenols in Sports Performance: A Holistic View

Polyphenols represent a sizeable heterogeneous class of compounds with common phenolic structural units present in nature in a broad array of foods such as fruits, vegetables, cereals, tea, chocolate, among others [40].

The various polyphenol groups are distributed according to the number of phenolic rings into flavonoids—more than 10,000 natural compounds—which can be further subclassified in many flavones, flavonols (e.g., from *Capparis spinosa*), flavanols or flavan-3-oils or catechins (e.g., from *Theobroma cacao*, *Camellia sinensis*) anthocyanins or anthocyanidins (e.g., from *Vaccinium myrtillus*), isoflavones and calcons (e.g., from *Glycine max*); and non-flavonoid polyphenols such as tannins, diferuloylmethanes (e.g., from *Turmeric Longa*), coumarins, benzophenones, secoiridoids, stilbenes (e.g., from *Polygonum cuspidatum*), phenolic acids, etc. [6,41,42].

In general, various health properties have been attributed to polyphenols, including antioxidant, anti-inflammatory, antibacterial, antiviral, antipruritic, antiparasitic, and cytotoxic [7,9,43,44,45,46].

To date, in athletic performance, numerous studies have investigated the antioxidant and anti-inflammatory potential of various polyphenols [47,48]. Few studies have described the genetic fingerprint of each athlete for their antioxidant capacity and anti-inflammatory response during exercise. People carrying specific genetic mutations (e.g., N-acetyltransferase (NAT)1/2, SOD1/2, glutathione peroxidase(GPX)1, paraoxonase(PON)1, x-ray repair cross-complementing family(XRCC)1) may have lower efficiency to modulate oxidative stress and inflammation during exercise and hence would require a significant increase in antioxidants with epigenetic mechanisms like polyphenols [49,50,51,52,53].

Very few studies have described the epigenetic role of polyphenols on the modulation of key proteins involved in energy balance, antioxidant mechanisms, glucose, and lipid homeostasis and the overall effect on sports performance.

One of the most innovative area to understand polyphenol health-related mechanisms in sports performance is the study of bidirectional interactions with the gut microbiota [10,40].

In plants, polyphenols are usually found in their glycosylated form, although esterified or polymerized forms may also be present [54]. Once ingested, polyphenols are recognized by the human body as xenobiotics, so their absorption ratio is remarkably lower than that of the nutrients introduced with the diet and varies greatly depending on the degree of polymerization or the complexity of their chemical structure. Only 5–10% of the polyphenols are absorbed in the small intestine, while the remaining 90–95% reaches the colon where it undergoes fermentation processes by the intestinal microbiota, and subsequently generate metabolites with different physiological implications. Following oral intake of 10 to 500 mg of polyphenols, the maximum plasma concentration generally does not exceed 1 μM, mainly due to poor absorption and metabolism by tissues and gastrointestinal microflora [40,54].

Polyphenols are also substrates for ATP-binding cassette (ABC) transporters, which are mainly efflux transporters, and which eliminate their substrates outside the cell. These proteins can influence the oral availability and tissue distribution of polyphenols, limiting their beneficial effects [55,56]. Genetic mutations affecting these transporters, such as those affecting hepatic and intestinal cytochromes, must be taken into account to determine the dosage of polyphenols based on the subject’s genotypic characteristics (poor, intermediate or extensive metabolizers) [57,58,59]. Interestingly, the interaction with the efflux transporters could be modulated by mixing the bioactive polyphenols with others so that the latter can be used to saturate the efflux transporter, thus increasing the oral bioavailability of the bioactive ones.

Once in the large intestine, polyphenols can both modulate the proliferation of specific bacteria and act as prebiotics for certain other microorganisms [60,61]. The International Scientific Association for Probiotics and Prebiotics (ISAPP) updated the definition of a prebiotic as “a substrate that is selectively utilized by host microorganisms conferring a health benefit”. This definition expands the concept of prebiotics to include non-carbohydrate substances, such as polyphenols [62]. A recent metanalysis showed that polyphenol supplementation enhances the abundance of *Lactobacillus* and *Bifidobacterium*, and reduces the abundance of some pathogenic *Clostridium* in the gut microbiota of the human subjects [63].

Understanding the final effect of polyphenols on human health thus requires a holistic view. The next section focuses on the use of polyphenols in sports performances by considering the genetic, microbiota and epigenetic influence on polyphenol effectiveness in order to customize doses and types of polyphenols for each athlete, improving sports performance.

### 3.1. Polyphenols and Sports Performance

#### 3.1.1. Curcumin

**Figure 2:**
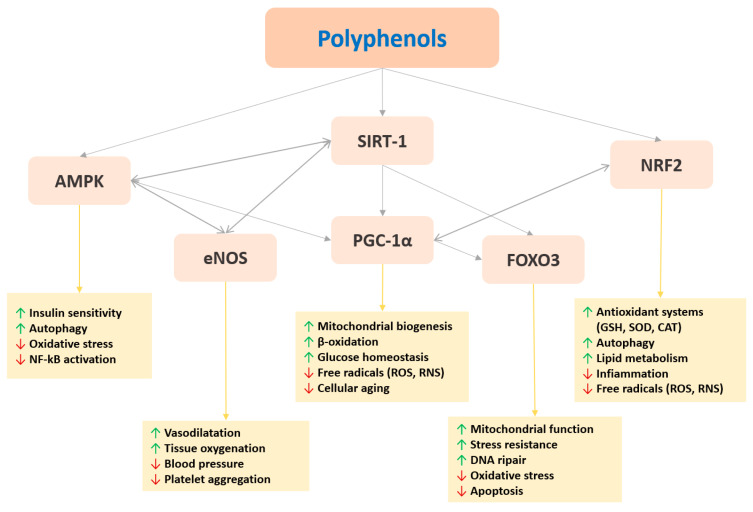
Main transcription factors modulated by polyphenols and related functions on sports performance. For figure description see the text. Legend: AMPK (5’ AMP-activated protein kinase); eNOS (endothelial nitric oxide synthase); SIRT-1 (NAD-dependent deacetylase sirtuin-1); NRF2 (Nuclear factor erythroid 2-related factor 2); PCG-1α (peroxisome proliferator-activated receptor gamma coactivator 1-alpha); FOXO3 (forkhead box O3); ROS (reactive oxygen species); RNS (reactive nitrogen species). NF-kB (nuclear factor kappa-light-chain-enhancer of activated B cells); GSH (glutathione); SOD (superoxide dismutase); CAT (catalase).

A prominent example of the use of polyphenols in sports nutrition is provided by curcumin. Curcumin (diferuloylmethane) is a component of the spice turmeric (*Curcuma longa*) phytocomplex, which has been part of the traditional Asian medicine for centuries. Curcumin has known anti-inflammatory, antioxidant, and anticancer properties. Numerous molecular signaling pathways for cell survival or modulation of transcription factors such as nuclear-related factor 2 (Nrf2) and nuclear factor kappa B(NF-κB) are regulated by curcumin. Curcumin is also an epigenetic modulator, acting on DNA methyltransferases (DNMT), microRNAs (miRNA), and histones acetylation/deacetylation (see Figure 2) [64].

In sports performance, the described properties of curcumin can prove useful as antioxidant and anti-inflammatory in processes related to muscle fatigue, muscle mass loss, muscle soreness, and post-exercise recovery [65]. Curcumin dosages vary from 40 mg to 6 g of curcumin (with an average dose of 80–200 mg of curcumin) per day or between 500 and 8000 mg per day of the whole turmeric standardized in curcuminoids (at least 90% of the extract) and curcumin [59,66,67,68,69,70]. Standardized extracts usually are more recommended as they contain both curcumin standardization and the whole phytocomplex that contributes to the beneficial actions, an information especially important when evaluating the possible interactions with the gut microbiota [69,70,71,72]. Athletes following high-calorie diets as in bodybuilding may experience a reduction in insulin sensitivity up to the development of insulin resistance, mainly related to a consistent increase in oxidative stress and low-grade inflammation with consequences in sports performance and muscle hypertrophy. In these athletes, curcumin supplementation can ameliorate redox homeostasis and insulin sensitivity through the modulation of inflammatory and oxidative pathways [73]. Curcumin may also be potentially useful to prevent muscle mass loss. By activating the transcription factor Nrf2, curcumin favors antioxidant defense and downregulates NF-kB activity, which is a crucial molecule in the path leading to muscle mass loss [73,74].

In response to stress factors, skeletal muscle can implement several adaptive responses, including mitochondrial biogenesis and clearance of damaged mitochondria, to promote muscle health. A recent study examined the effects of curcumin and resistance training in mitochondrial biogenesis in ten-week-old male Wistar rats. The results showed an increase of various parameters involved in mitochondrial biogenesis in skeletal muscle (oxidative phosphorylation(OXPHOS) subunit, mitochondrial DNA copy, AMP-activated protein kinase(AMPK) phosphorylation, the NAD(+)/NADH ratio, sirtuin(SIRT)1 expression, peroxisome proliferator-activated receptor gamma coactivator(PGC)1α deacetylation, cAMP levels) [75].

In 2017, Delecroix et al. analyzed the daily effect of supplementation with 2 g of curcumin and 20 mg of piperine, 3 times a day, each day between 48 h before and 48 h after exercise-induced muscle damage in ten elite rugby players. Curcumin and piperine supplementation showed a partial impact on some aspects of muscle recovery. The main results showed a moderate effect in favor of curcumin, and piperine supplementation on the loss of one leg 6 s sprint mean power output 24 h after the exercise (ES = −1.12; CI90% = −1.86 to −0.29). When the recovery period between competitions was short, curcumin and piperine supplementation could be an effective recovery strategy to attenuate muscle damage. Moreover, the improvement in sprint mean power output was moderately faster in the curcumin and piperine group [76].

Occasional exercise can cause muscle damage and consequent reduced physical performance, especially if it is eccentric. Downhill running is an experimental model used to cause muscle damage and induce oxidative stress and inflammatory reaction. An in vivo study in mice showed that curcumin reduces inflammatory cytokine concentrations in skeletal muscle by modulating oxidative stress following muscle damage caused by downhill running [77].

The use of curcumin to alleviate muscle damage has also been evaluated in a double-blind, randomized, placebo-controlled study in 63 physically active people, showing an improvement in physical performance and recovery. 200 mg curcumin given for eight weeks attenuated muscle soreness down to 74%, 80%, and 92% immediately, 24 h, and 48 h after exercise, respectively and improved some performance parameters such as peak flexion torque, which was significantly reduced already after one hour (95% CI: 1.42–6.36, *p* = 0.04), at the end of the downhill running in some subjects [68].

Delayed onset muscle soreness (DOMS) due to eccentric muscle activity is associated with inflammatory response and production of reactive oxygen species (ROS). This inflammatory state is necessary to implement muscle repair mechanisms, but if excessive and perpetrated over time can exacerbate muscle damage and catabolism, especially in older subjects [78]. Curcumin seems a promising molecule to mitigate muscle damage induced by oxidative stress and inflammation in continuous eccentric exercise.

A randomized, placebo-controlled, single-blind pilot study with twenty healthy moderately active males, was conducted to investigate whether 1 g twice daily Phytosome^®^ release system (200 mg curcumin b.i.d.) could reduce the extent of DOMS in terms of both pain intensity and muscle injury. Subjects in the curcumin group reported less pain in the lower limbs than subjects in the placebo group, although the analysis did not reach statistical significance (total score: 23.3 ± 7.9 vs. 30.6 ± 7.9, *p* = 0.06). The increases in muscle damage and inflammation markers tended to be lower in the curcumin group with significant differences for interleukin-8 (IL-8) (*p* < 0.05) at 2 h after exercise. No differences in oxidative stress markers and muscle histology were observed [59].

Another work assessed the effect of curcumin in sedentary subjects in exercise-induced muscle damage (EIMD) and DOMS, parameters that impact subsequent training sessions and activities of daily living even on active individuals. In sedentary or diseased individuals, EIMD and DOMS may be more pronounced and occur even in the absence of structured exercise. Curcumin supplementation resulted in a significantly smaller decrease in CK (−48%; *p* = 0.035), TNF-α (−25%; *p* = 0.028), and IL-8 (−21%; *p* = 0.030) following EIMD compared to placebo, thus supporting the use of oral curcumin supplementation to reduce the symptoms of EIMD [79]. Further evidence suggests that oral curcumin likely reduces pain associated with DOMS and enhances recovery of muscle performance [80,81]. Preliminary in vivo results have described curcumin actions on muscle strength and fatigue during exercise. Huang et al. evaluated the potential beneficial effects of curcumin supplementation on fatigue and ergogenic function in mice under physical exertion for four weeks [82]. The levels of biomarkers associated with physical fatigue, ammonia, blood urea nitrogen (BUN), and markers of glucose and tissue damage such as aspartate transaminase (AST), alanine transaminase (ALT) and creatine kinase(CK) were monitored. Curcumin supplementation had a dose-dependent effect on increasing strength and endurance, significantly reducing lactate, ammonia levels, BUN, AST, ALT and CK after a physical challenge. A significant increase in muscle glycogen levels was also observed [82].

One of the most discussed issues regarding curcumin is related to its poor bioavailability, for which many companies have made liposomal, phytosomal, or micellar preparations to increase its absorption [83]. Although these formulations can be useful in conveying curcumin within the body, most of the curcumin benefits are probably related to its interaction with the intestinal microbiota. Curcumin and its derivatives exert direct regulatory effects on the intestinal microbiota, which could explain the paradox between the low systemic bioavailability and its widely reported pharmacological activities [84,85]. In in vivo studies, curcumin has been shown to modulate the *Firmicutes*/*Bacteroidetes* ratio, thus modifying the colon’s microbial ecology, and at the same time to exert anti-inflammatory and anti-carcinogenic effects [86,87].

Curcumin can significantly improve the relationship between beneficial microbiota and pathogens by increasing the abundance of bifidobacteria, lactobacilli, and butyrate-producing bacteria. Moreover, curcumin reduces the proliferation of enterococci, coriobacterales, and enterobacteria and promotes the integrity of the intestinal barrier by exhibiting an immunomodulatory and anti-inflammatory action [86,88,89,90,91].

In conclusion, curcumin can be considered an effective natural remedy to modulate oxidative stress and inflammation, improving athletic performance. However, more extensive studies are required to confirm these results and further clarify the mechanism of action of curcumin in sports performances, considering the bidirectional influence of curcumin and the gut microbiota. In terms of practical applications, curcumin supplementation must be evaluated according to the type of formulation. The standardized extract of turmeric can be used daily for its primary prebiotic effect on the intestine. In contrast, high bioavailability curcumin formulations such as micellar, liposomal, phytosomal, among many others, should be used with more caution for about 3-month cycles, under a health expert’s supervision.

#### 3.1.2. Resveratrol

Resveratrol (3,5,4′-trihydroxy-stilbene) is a natural phytoalexin (from the Greek *aléxein* = to guard or protect), produced as a stress-signaling molecule by plants, such as *Polygonum cuspidatum*, in response to changes in the environment or following nutrient deficiency, both to defend against dangerous environmental situations (UV rays, pathogens, etc.) and to implement animal early defensive interventions to survive [92,93,94].

Resveratrol exists in two geometric isomers, in which the two phenolic rings are arranged in the *trans* or *cis* configuration. Although plants in nature produce both forms, the *trans* form seems the most stable and biologically active [95,96].

There are numerous documented pharmacological activities of resveratrol on humans, but its antioxidant, and anti-inflammatory activities are the best characterized [97]. Resveratrol can activate one of the members of the sirtuin family, SIRT1, in concert with PGC1α inducing its deacetylation and therefore increasing its transcriptional activity on multiple proteins involved in mitochondrial biogenesis, insulin regulation, lipid, and energy metabolism (see Figure 2) [98,99].

Many studies have provided evidence of neuroprotective, antiatherogenic, antithrombotic, antihypercholesterolemic, vasorelaxants, and anticancer properties of resveratrol [95,100,101,102,103].

The average resveratrol daily dose varies from 100 to 1000 mg of *trans*-resveratrol or between 250 and 5000 mg per day of different resveratrol sources, such as a standardized extract of *Polygonum Cuspidatum* [104,105,106,107]. It is essential to consider that not all extracts have the same *trans*-resveratrol standardization, although plants also contain glycosylated precursors of resveratrol, including polydatin [108].

In exercise, resveratrol appears to improve muscle strength and fatigue tolerance, and muscle regeneration after disuse [48,109,110]. Bennet et al. demonstrated the effect of resveratrol in enhancing muscle mass after disuse in aging [111]. In old mice, resveratrol primes the impacts of physical activity, increasing mitochondrial functionality and providing ergogenic mechanisms to maintain muscle performance during aging [112].

In 2016, Polley et al. assessed skeletal muscle mitochondrial capacity on sixteen healthy young adults who received either placebo or 500 mg of resveratrol plus 10 mg piperine randomly for four weeks. In the untrained group, neither the placebo nor the resveratrol plus piperine increased mitochondrial capacity. Nevertheless, during low-intensity exercise training resveratrol plus piperine significantly increased skeletal muscle mitochondrial capacity (*p* = 0.02) [113], thus showing an exercise-dependent effect of resveratrol [114].

In sports performance, bodyweight management, targeted body fat reduction, and increased lean body mass are of significant importance. Resveratrol has shown both in vivo and human studies to possess a plethora of ergogenic, hypoglycaemic, and anti-obesity properties useful for maintaining an optimal body composition [115,116,117].

At the molecular level, resveratrol can stimulate PGC-1α and AMPK, among others, which are involved in fatty-acid beta-oxidation and glucose metabolism [118].

Sun et al. showed that exercise combined with resveratrol supplementation in obese mice exhibited anti-obesity effects in the long term due to enhanced mitochondrial biogenesis [119].

In a randomized, double-blind crossover study of 11 healthy and obese men, the metabolic effects of resveratrol 150 mg/day for 30 days were evaluated. Resveratrol stimulated AMPK, SIRT1 and PGC-1α protein levels, and citrate synthase activity by improving mitochondrial muscle respiration. In the liver, there has been an improvement in triglycerides, inflammation markers, and intrahepatic lipid content. A blood pressure lowering and homeostasis model assessment insulin resistance (HOMA-IR) index effect was also observed. Overall, resveratrol supplementation induced protective metabolic changes in obese subjects [117]. This preliminary evidence may be translated into athletes to improve body composition and sports performance, although further studies are required.

Another important aspect of resveratrol supplementation in sports performance is the effect on glucose control and insulin sensitivity. The latter is a new hot topic in bodybuilding, and there are some very valid reasons for that. In fact, one of the most critical physiological tasks during a physical transformation is making the body use insulin as much efficiently as possible, and resveratrol seems to be a promising molecule to improve this. A meta-analysis of 11 randomized controlled trials aimed to quantitatively evaluate the effects of resveratrol on glucose control and insulin sensitivity. Resveratrol significantly improved glucose control and insulin sensitivity in diabetic or prediabetic subjects without altering glycemic measures in nondiabetic individuals [100,120].

Taken together, these results suggest that resveratrol may be useful for those athletes who have hyperglycemic fluctuations, and insulin resistance to improve insulin actions in muscle absorption and growth.

The potential beneficial effect of resveratrol in sports performance must nowadays consider the interaction between this polyphenol and the intestinal microbiota. As for other polyphenols, several studies have shown that the interaction between resveratrol and intestinal microbiota is bidirectional and that this is associated with multiple metabolic effects of significant importance for human physiology [87,118,121].

The intestinal microbiota performs the first important biotransformation of resveratrol by metabolizing it into dihydroxy-resveratrol. At the same time, two specific bacterial strains *Slackia equolifaciens* and *Adlercreutzia equolifaciens* produce the further two metabolites 3,4′-dihydroxy-trans-stilbene and lunularin [118].

Resveratrol’s impact over the gut microbiota is associated with changes in body weight and body fat, as well as improvement in glucose homeostasis and obesity-related parameters. The effects are achieved both through the promotion of some bacteria related to energy metabolism and through the action of its gut-microbiota-produced metabolites, which in turn modulate biochemical pathways related to energy metabolism [118]. Furthermore, resveratrol effectively improves the growth of *Lactococcus lactis* and *Akkermansia muciniphila.* The latter is a Gram-negative, obligate anaerobic eubacterium, classified under the phylum Verrucomicrobia that stimulates the production of mucous on the gut lining, strengthens gut barrier and helps control glucose metabolism and inflammation [122]. Finally, resveratrol also inhibits the proliferation of *Enterococcus faecalis* and reduces the production of trimethylamine from choline by remodeling the intestinal microbiota’s composition [87,118,121].

These new findings need to be considered in future studies evaluating resveratrol’s actions in sports performance in order to better understand the role of this molecule in a systemic way.

#### 3.1.3. Cocoa Flavanols

In recent years, a vast amount of research has focused on the beneficial effects of flavanols, a class of polyphenols, present in many types of food, including cocoa, tea, vegetables, and fruits. The large family of flavanols or flavan-3-ols—which includes catechins, epicatechins, and their oligomers such as those present in cocoa, have been shown to possess numerous beneficial properties.

Cocoa (*Theobroma cacao* L., 1753; from Nahuatl: cacahuatl) is a typical evergreen tree belonging to the Sterculiaceae family originally from central and south America and swiftly spread out to other parts of the world [123]. The ancient Central and South American populations used cocoa as aphrodisiac, but also energizer and to cure certain diseases [124].

Named after this plant, theobroma (“food of the gods”) cocoa beans contain the xanthine theobromine in significant quantity, as well as flavonols and nearly 400 other identified molecules. Cocoa flavanols are modulators of oxidative stress and inflammatory processes. They also induce the production of nitric oxide (NO) with consequent vasodilation, improvement of endothelial function, and reduction of blood pressure. An improvement in insulin sensitivity and lipid profiles was also shown in subjects with or without cardiovascular risks [125]. At the brain level, cocoa flavanols, once crossed the blood-brain barrier, act by increasing the cerebral blood flow and promote cognitive functions [126]. In recent years, with the advent of studies on intestinal microbiota, it is becoming clear that a large part of cocoa polyphenols undergo extensive biotransformation in the large intestine by the resident commensal bacteria. It is also gradually being understood that the diversity and specificity of these microorganisms are essential for polyphenol metabolism to produce small secondary metabolites that probably represent the bioactive compounds interacting with human biochemical pathways [127]. Two main metabolites, phenyl-γ-valerolactone, and phenylvaleric acid are found in the urine after 5–10 h from oral intake of dark chocolate or green tea [128,129,130].

What is even more interesting and less known is cocoa polyphenol influence on the intestinal microbiota diversity. Out of the many gut microbial species, *Escherichia coli*, *Bifidobacterium* sp., *Lactobacillus* sp., *Bacteroides* sp., *Eubacterium* sp., are known to be mainly responsible for the cocoa’s polyphenol metabolism [40]. Cocoa polyphenols appear to modulate microbial diversity by promoting the proliferation of some bacteria and inhibiting other potentially pathogenic ones. These actions can fully be ascribable to prebiotic mechanisms, as reported by the ISAPP [41,62]. The effect of cocoa flavanols on sports performance is controversial. In 2018, a systematic review by Decroix et al. evaluated the effects of cocoa flavanols on exercise performance and recovery and exercise-induced changes in vascular function, inflammation oxidative stress, cognitive function, and metabolic markers in humans. Thirteen studies, with a total of 240 participants, were analyzed. Although the author concluded that further studies were needed, the results showed that cocoa flavanol supplementation improves vascular function, reduces exercise-induced oxidative stress, and alters fat and carbohydrate utilization during exercise, without affecting athletic performance [131].

Further, in a randomized, double-blinded design, 13 male collegiate rugby players were evaluated while consuming either chocolate milk or chocolate milk with additional cocoa flavanols during a seven day loading phase. No changes in oxidative stress or athletic performance benefits were observed between the groups over time [132]. In another randomized, double-blind study, 14 well-trained male cyclists were examined for the effects of seven day cocoa flavanol intake on oxidative stress, nitric oxide production, and tissue oxygenation in response to the exercise of normobaric hypoxia. The results showed that cocoa flavanol supplementation had beneficial effects on endothelial function at rest, as well as on prefrontal oxygenation at rest and during moderate-intensity exercise, both in normoxia and hypoxia. In addition, cocoa flavanols inhibited oxidative stress during intense hypoxic training [133].

Although controversial, most scientific evidence highlights the beneficial role of cocoa flavanols on sports performance. The differences observed are probably related to genetic variables and the diversity of the intestinal microbiota in each individual, which can impact cocoa flavanol metabolism and their beneficial effects.

Even for cocoa flavanols, as for the other polyphenols, it is necessary to clarify the interactions with the intestinal microbiota to understand the mechanisms at the basis of multiple beneficial effects exerted by these molecules, especially at the cardiometabolic and psycho-cognitive level [134,135]. Cocoa polyphenols are absorbed only minimally in the small intestine as monomeric or dimeric forms. At the same time, most of them, reach the colon and undergo metabolism by the intestinal microbiota to form small bioactive molecules that carry out their beneficial properties once the biological target is reached in the body [128,136]. Out of the many gut microbial species, *Escherichia coli*, *Bifidobacterium* sp., *Lactobacillus* sp., *Bacteroides* sp., *Eubacterium* sp., are known to be mainly responsible for the cocoa’s polyphenol metabolism [40].

In a double-blind, randomized controlled clinical trial, twenty-two healthy human volunteers were randomly assigned to a high cocoa flavanol group (494 mg cocoa flavanols/d), or a low cocoa flavanol group (23 mg of cocoa flavanols/d) for four weeks. The results showed that the consumption of cocoa flavanols could significantly influence the growth of selected intestinal microflora in humans, mainly bifidobacteria and lactobacilli. On the other hand, a significant reduction of Clostridia was seen. These changes in the bacterial population have been linked to changes in other biological markers such as triglycerides and C-reactive proteins, which are markers of metabolic problems and inflammation [137]. This study suggests the potential prebiotic benefits associated with the dietary inclusion of foods rich in flavanol and which can, in a translated way, be also useful in athletes.

Today the tipical daily doses of cocoa flavanols vary from 5 to 1000 mg of flavanols with an average between 200–500 mg flavanols contained in different formulations, such as cocoa powder, flavanol-enriched dark chocolate, cocoa bars, or others flavanol enriched products usually taken before sports performance [131,138].

#### 3.1.4. Quercetin

Quercetin is a naturally occurring polyphenol in many fruits and vegetables, tea, and berries. It belongs to the flavonol family (tetraoxyflavonol), and is the aglyconic component of various glycosides, including rutin and isoquercitrin [139].

As with the other polyphenols, the bioavailability of quercetin is strongly influenced by intestinal absorption and by its interaction with the microbiota depending on its aglyconic or glycosylated form.

In the small intestine, glycosylated quercetin (isoquercitrine or rutin) is deglucosylated in aglycone by lactase-phenorizin hydrolase (LPH) or cytosolic β-glucosidase (CBG) of intestinal epithelial cells, and then converted to O-glucuronide/O-sulfate quercetin by intestinal cell phase II enzymes. Genetic polymorphisms affecting some efflux pumps such as breast cancer resistance protein(BCRP) can limit quercetin absorption in the enterocyte. The use of other concomitant polyphenols, known inhibitors of these efflux transporters, can promote the absorption of quercetin. For example, quercetin bioavailability can be significantly increased when co-consumed with a polyphenol BCRP inhibitor-like apigenin or hesperetin [140]. Once absorbed, these conjugated metabolites are transported to the liver, where they are further metabolized before entering the systemic circulation or being re-directed into the intestine through the enterohepatic circulation. Glycosylated quercetin has a maximal concentration higher than quercetin as an aglycone.

Moreover, rutin (quercetin-3-O-β-rutinoside) is poorly absorbed in the small intestine and it reaches the large intestine, where it interacts bidirectionally with the colon microbiota which degrades rutin to form quercetin, which can then be absorbed as such or undergo further degradation by enterobacteria to produce other metabolic products [10]. In the colon, some types of bacteria can degrade rutin into metabolites, including 3,4-dihydroxyphenylacetic acid, 3-hydroxyphenylacetic acid, and 3-(3-hydroxyphenyl) propionic acid thanks to the production of enzymes known as quercetin dioxygenases, or quercetinase. The enzymatic cleavage reactions of glycosylated or aglyconic quercetin require oxygen. In the anoxic environment of the colon, anaerobic bacteria such as Bacillus subtilis carry out these enzymatic processes. As the primary intestinal microbial metabolite of quercetin, protocatecuic acid contributes to beneficial health effects [10].

Numerous in vitro and in vivo studies testify to the antioxidant, anti-inflammatory, and cardioprotective effects of quercetin [139,141]. Quercetin appears to modulate uricemia, thereby reducing the risk of cancer and other chronic diseases. It has also been shown to improve mental and physical performance, as well as immune system functions thereby reducing the risk of infection during intense exercise [142,143,144].

The mechanisms suggested for the plethora of beneficial effects of quercetin on sports performances are many. Quercetin has been shown to interferes with the inflammatory mechanisms induced by lipopolysaccharides (LPS), modulating toll-like receptors (TLRs) activation, and reducing the production of TNF-a, interleukin(IL)1a in macrophages and glial cells. It also modulates the synthesis and activity of enzymes such as cyclooxygenase (COX) and lipoxygenases (LOX) in inflammatory processes. The immuno-stimulating effect of quercetin appears to be related to epigenetic mechanisms on interferon(IFN)γ and IL-4 production. Quercetin induces gene expression and (IFN-γ) production, which promotes type 1 T helper (Th1) response and down-regulates IL-4) for Th2 response from normal peripheral blood mononuclear cells. Quercetin, therefore, can influence the immune response and the mechanisms of inflammation by acting through multiple molecular mechanisms, some of which are also linked to the modulation of enzymes and membrane proteins and intracellular signaling kinases and phosphatases. Quercetin can also boost both physical and mental performance and increase mitochondrial biogenesis in rodent muscle and brain [143].

In 2017, a systematic review and meta-analysis aimed to determine the overall effect of polyphenols on human athletic performance. Polyphenols supplementation for at least seven days resulted in increased athletic performance by 1.90% (95% CI 0.40–3.39), with a 2.82% increase in performance (95% CI 2.05–3.58) for quercetin. Overall, polyphenols, and in particular quercetin, are considered useful supplements to improve performance in healthy subjects with no reported side effects [145].

In humans, most studies on quercetin action in athletes have been focused on phenotypic mechanisms related to sports performance such as sprint and endurance exercises, resistance training, or sports competitions such as marathon or triathlon [145]. A double-blind clinical study of 60 male students with an athletic history of at least three years, evaluated the effect of supplementation of 500 mg of quercetin daily for eight weeks on body composition, physical performance, and some blood biomarkers. At the end of the eight weeks, basal metabolic rate (*p* = 0.001), lean body mass (*p* ≤ 0.001), total body water (*p* ≤ 0.001), and overall energy expenditure (*p* = 0.001) increased significantly in the quercetin group, suggesting that quercetin supplementation ameliorate some performance parameters in athletes [146].

Another randomized, double-blind study evaluated the effects of 1 g/day of quercetin for six weeks in 8 young adult long-distance runners on endurance performance and antioxidant status and found a reduction in peroxidation levels (*p* = 0.019) without significant performance changes (*p* = 0.172) [147].

Ten young men participated in a double-blind, randomized crossover study to examine the acute effects of 1 g quercetin on neuromuscular function, muscle damage biomarkers, and rate of perceived exertion in response to a single resistance training session. Quercetin improved the torque-velocity curve of knee extensors. After resistance exercise, maximum voluntary isometric contraction was less reduced with a higher rate of torque development and neuromuscular efficiency ratio. The total volume of resistance exercises was significantly higher than in the placebo group. In this study, acute ingestion of quercetin improved neuromuscular performance during and after a resistance training session [148].

In another randomized, double-blind, crossover study, a reduction in strength loss and neuromuscular impairment associated with eccentric exercise-induced muscle damage was assessed in twelve young men who received 1 g/day of quercetin or placebo for 14 days. Fourteen days of quercetin intake significantly increased the isometric strength in maximal voluntary isometric contraction (MVIC), compared to baseline (+4.7%, *p* < 0.05) and attenuated muscle weakness severity caused by eccentric-induced myofibrillar disruption and sarcolemmal action potential propagation impairment [149].

High oxidative stress can affect muscle recovery and post-workout pain sensation in athletes. In a pilot registry study, the effects of quercetin phytosome^®^ supplementation in amateur triathlon athletes were determined. 250 mg quercetin phytosome^®^ twice a day in a total of 23 subjects showed an improvement in the time to complete the run. Likewise, post-stroke muscle pain, localized pain, oxidative stress, cramps, and post-exercise recovery time eased [150].

Overall, quercetin represents a valid sports supplement able to regulate multiple pathways related to sports performance and post-race recovery. Interindividual variations at the genetic level and in the composition of the gut microbiota can affect quercetin metabolism and should be considered to recommend a personalized dosage for each individual.

Currently, the daily dose of quercetin typically used in human studies ranges from 50 to 2000 mg per day, with an average daily dose of 200–1000 mg, depending on the formulation and bioavailability [151,152,153].

#### 3.1.5. Other Polyphenols

Although quercetin is the most studied flavonoid for its beneficial effects in sports performance, numerous other molecules in the world of polyphenols seem promising in influencing physical performance and reducing fatigue and pain after exercise.

Green-tea extracts, rich in catechins, have promising effects in improving recovery from exercise thanks to their antioxidant, anti-inflammatory, and lipid metabolism regulatory properties [154].

In sports performances, supplementation with an average dose from 250 to 1000 mg per day of green tea extracts before the event reduces muscle damage and oxidative stress with positive effects on neuromuscular parameters on muscle fatigue [154,155,156,157,158]. A double-blind, randomized, placebo-controlled crossover study of 16 sprinters measured the impact of a green tea extract (GTE) (980 mg polyphenols per day) on SOD activity, glutathione peroxidase, total polyphenols, total antioxidant capacity (TAC), uric acid (UA), albumin (AL), malondialdehyde (MDA) and CK as well as an effect on sprint performance. The results showed that supplementation with GTE prevented repeated cycle sprint (RST)-induced oxidative stress in sprinters, without hindering training adaptation in the antioxidant enzyme system. However, the administration of GTE did not prevent exercise-induced muscle damage, nor did it improve sprint performance [154]. An interesting study analyzed the effect of 500 mg/day of GTE on sixteen male amateur athletes trained for 15 days. The effects of integration were tested during repeated tests of the submaximal cycle at 60% of the peak power, supplied after a protocol for cumulative fatigue of the knee extensors. GTE supplementation reduced the magnitude of muscle damage and oxidative stress in response to fatigue, with positive effects on neuromuscular function in response to a condition of cumulative fatigue, with respect to placebo [157]. Therefore, the use of green tea extracts rich in catechins can prove to be a valid strategy in reducing exercise recovery time.

Polyphenolic blueberry extracts have shown antioxidant, anti-inflammatory, and vasodilator effects in several human studies on improving recovery from exercise [159]. Moreover, numerous prospective studies show that the intake of anthocyanin-rich blueberries is associated with a reduction in the risk of type 2 diabetes and cardiovascular disease. A randomized, double-blind, six month study examined the effect of consumption of blueberries (75–150 g/day) on 115 subjects of average age 63 years, for the evaluation of insulin resistance and cardiometabolic functions. A daily intake of one cup of blueberries improved endothelial function, systemic arterial stiffness, and increased HDL cholesterol levels, thus suggesting that blueberries could be included in dietary strategies to reduce the risk of cardiovascular disease [160]. In a randomized cross pattern, 10 women consumed a blueberry smoothie or a placebo with similar antioxidant capacity. Blueberry smoothie ingestion before and after the EIMD improved isometric strength recovery of the muscle peak. This effect seemed to depend on upregulation of adaptive processes, such as endogenous antioxidant systems, activated by the combined actions of eccentric exercise and blueberries consumption regardless of intrinsic antioxidant capacity [161]. The protective effect of blueberries consumption might thus be mainly related to indirect mechanisms linked to interactions with the microbiota [162,163]. Once in the gut, blueberry polyphenols such as proanthocyanidins tend to reduce the Firmicutes/Bacteroidetes ratio and increase the relative abundance of *Akkermansia muciniphila*. This can increase beta-oxidation of fatty acids and browning of white adipocytes, and at the same time reduce inflammatory states associated with the metabolic syndrome [164,165,166,167]. Ellagitannins, vice-versa, can strongly inhibit the growth of pathogenic bacteria such as *Clostridium* and *S. aureus*, favoring the proliferation of bifidobacterial [168], while tannic acid can favor the growth of *Lactobacillus acidophilus* [169]. The reshaping of the microbial diversity could be the main event responsible for the beneficial properties of blueberries on health and sports performance. Further studies are underway to further evaluate their effectiveness.

The French maritime pine bark extract rich in oligomeric proanthocyanidins (OPC), Pycnogenol^®^, has shown promising properties in sports performance. Preliminary studies have reported that supplementation with Pycnogenol^®^ at dosages between 100–800 mg/day can improve physical performance and protect from post-exercise oxidative stress, both in normal subjects and athletes [170,171].

A new and promising source of polyphenols for sports performance is the french Montmorency cherry concentrate (*Prunus cerasus*). It is used as a food supplement and has a high concentration of anthocyanidins. Numerous studies have highlighted potent anti-inflammatory and antioxidant properties, as well as the ability to promote immunity, sleep, muscle recovery, and reduce post-exercise pain, mainly in strength sports [172,173,174,175]. A 2010 study on twenty recreational marathon runners found that taking cherry juice for five days reduced post-run inflammation, compared to placebo, and isometric strength recovered faster (*p* = 0.024) [176].

In a different study on 16 trained cyclists, 30 mL of Montmorency cherry juice concentrate, administered twice a day for seven days, induced a greater reduction in inflammation and oxidative stress markers (lipid hydroperoxides (LOOH), *p* < 0.01; IL-6, *p* < 0.05 and high-sensitivity C-reactive protein (hsCRP), *p* < 0.05), suggesting its use as a supplement during multi-day races [173]. Moreover, a crossover design study on 10 trained cyclists showed that taking 30 mL of cherry tart juice 1.5 h before driving at high intensity until exhaustion, improved 9.5% of their peak power during a full 60-s sprint, suggesting a benefit on performance [177].

Finally, the acute supplementation of 40 mg of Ecklonia cava polyphenols (ECP), brown algae from Japan and Korea, was assessed on twenty collegiate students aged between 18 and 23 years. ECP was compared to placebo, 30 min before each exercise test, to analyze the maximum endurance capacity and related physiological parameters [178]. ECP supplementation increased depletion time (2.39 min), and at the same time mean VO2max was found 6.5% higher, compared to placebo. Furthermore, 3 min after exhaustive exercise, blood glucose levels in the ECP group were significantly higher (+9.9%), and the post-exercise blood lactate levels slightly reduced. The authors concluded that ECP supplementation might contribute to greater glucose metabolism and lower lactate production during intense exercise, probably due to anti-radical and enhanced circulatory activities [178].

### 3.2. Polyphenol Epigenetic Mechanisms and Sports Performance: A Roadmap for Future Practical Applications

Epigenomics is the study of the complete set of epigenetic modifications on the genetic material of a cell, known as the epigenome [179]. Epigenetic changes are reversible modifications on a cell’s DNA structure or histones that affect gene expression without altering the DNA sequence. DNA methylation, histone modifications, chromatin remodeling, non-coding RNA are deeply interconnected layers, all playing a role in epigenomic modifications that ultimately affect DNA expression [180].

The heritability of specific phenotypical traits is relevant for physical performance, but modulation of gene expression, mainly through DNA methylation and histone modifications, leads to persistent effects on the availability of DNA for transcription [181,182]. With the exceptions of genomic imprinting and some documented epigenetic inheritance, epigenetic changes are thought not to be inherited transgenerationally [182]. Along with their susceptibility to external influences, epigenetic patterns are highly specific to the individual and may represent pivotal control centers predisposing towards higher or lower physical performance capacities [182,183]. Epigenetic modifications alter accessibility to DNA and change chromatin structure, thereby regulating gene expression patterns. Methylated histones can act as binding sites for certain transcription factors or prevent the binding of transcription factors by hiding the recognition site [184]. There are growing evidence indicating that remodeling of the human epigenome constantly continues throughout life through nutrition, exercise, mental pressure or stress, and environmental stimuli [185,186].

Among epigenetic modulators, exercise itself plays a fundamental role. Epigenetic mechanisms affected by physical activity are known to be involved in metabolic, cognitive, and age-related processes. In general, even light exercise can induce hypomethylation of the entire genome within muscle cells, activating many regulatory genes involved, for example, in repair and muscle growth [184,187].

Molecules with epigenetic effects can potentially be used to guide the epigenome functions and thus modulate DNA expression, by acting on one or more of the players of the “epigenetic orchestra” and their mutual interactions, thus achieving genotype-phenotype interactions of therapeutic interest [183,188]. In sports nutrition, epigenomic approaches could be applied to reduce muscle inflammation, to faster muscle recovery, or to stimulate endogenous antioxidant systems [182,188].

An important family of epigenetic modulators is represented by plant polyphenols [189,190,191]. Plants produce polyphenols as secondary molecules in response to stressful situations [192,193]. Once ingested by other organisms belonging to the animal world or adsorbed by mushrooms, polyphenols stimulate survival pathways mediated by proteins called sirtuins, which, at least, in fungi and small animals, can prolong the lifespan [92]. The “xenormetic hypothesis”, coined by Howitz and Sinclair in 2003 [98], suggests how animals and fungi perceive stress-signaling molecules produced by other species, such as plant polyphenols, in response to environmental changes or nutrient deficiency to survive.The xenormetic phenomenon is the basis of the epigenetic mechanisms induced by plant polyphenols. Epigenetic modulation by natural compounds such as polyphenols has become a hot topic in healthy aging and sports performance, especially for personalized approaches. Polyphenols are known modulators of the epigenetic state of cells and can reverse abnormal or altered gene expression patterns [186,194,195]. Plant polyphenols modulate oxidative stress and inflammation and regulate metabolic and energy pathways that can be translated into stable epigenetic models of gene expression. The complex interactions between polyphenols and histone modifications, DNA methylation, the expression of non-coding RNA, and chromatin remodeling factors can influence the inflammatory phenotype by reducing the low-grade inflammation states that can predispose an individual to develop chronic pathologies [191,196].

Epicatechins present in cocoa or green tea, have been shown to restore the expression of antioxidant enzymes through demethylation of the promoter of glutathione-S-transferase P1 (GSTP1) and inhibition of DNA methyltransferase family (DNMT) [197,198,199]. Furthermore, a cocoa extract has been shown to inhibit the expression levels of the genes encoding DNMT and methylenetetrahydrofolate reductase (MTHFR) in vitro [195,200]. In a study with 214 volunteers with cardiovascular risk factors, who were in pre-hypertensive, stage-1 hypertensive, or hypercholesterolemic conditions, the epigenetic effects of 6 g/day cocoa for two weeks were evaluated. The study found a decrease in the global DNA methylation of peripheral blood leukocytes, suggesting a beneficial effect of cocoa [195].

To date, the evidence relating to the epigenetic effects of polyphenols on sports performance is in its early steps, and most knowledge is extrapolated from other studies on inflammation, cancer, or oxidative stress. Certainly, these preliminary findings are extremely promising and are opening new perspectives for future developments of personalized interventions in sports performance.

## 4. Conclusions and Future Perspectives

Dietary polyphenols exert multiple beneficial effects on sports performance demonstrated in both in vivo and human studies. Polyphenol health-related mechanisms mainly concern the modulation of mitochondrial biogenesis and the stimulation of enzymes or transcription factors related to stress, as well as a nutritional deficiency (NRF2, PGC1α, forkhead box(FOXO)3, AMPK, Sirt1), that regulate gene expression of key antioxidant proteins (SOD, Catalase, Glutathione system, etc.) (Figure 2). They also have been shown to modulate inflammatory processes (Nf-KB, COX, LOX, etc.) and the immune system response (Th1/Th2 balance). Furthermore, some polyphenols favor vascular regulation and endothelial function in humans by increasing endothelial nitric oxide synthesis.

**Figure 3:**
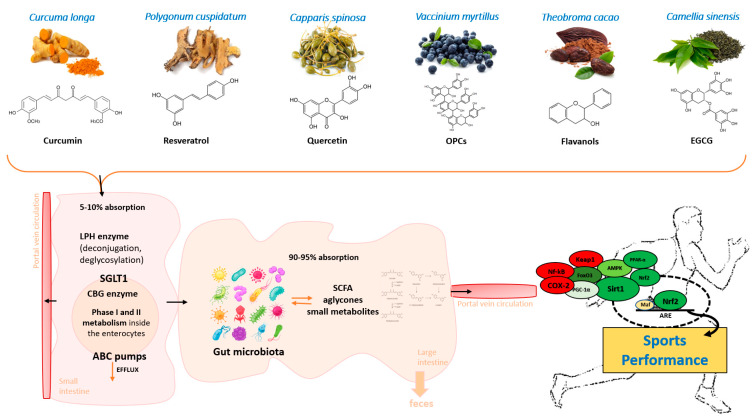
Polyphenol bioavailability and the influence of the gut microbiotaOnce ingested, dietary polyphenols’ metabolism begins in the oral cavity. In saliva, glycosylated flavonoids can be hydrolyzed into aglycones and then converted into smaller compounds, which are subsequently absorbed by the oral epithelium [201]. Once in the stomach, some polyphenols undergo a first reduction in monomeric units [10] and can exert direct protective effects on the gastrointestinal tract [202]. Then, the small intestine is responsible for the absorption of a low amount of polyphenols, mainly after de-conjugation reactions such as de-glycosylation. Aglycones can be absorbed directly in the small intestine, while glycosides, esters, and polymers typically require a first hydroxylation by the intestinal enzymes of the small intestine or by the colon microflora before absorption [203,204]. Unabsorbed polyphenols continue their path in the colon, where the intestinal microbiota continues the metabolic processes. In addition, polyphenols absorbed in the upper part of the gastrointestinal system—metabolized by the liver and excreted in the bile or directly extruded by the efflux pumps of the small intestine enterocytes—reach the colon and experience microbial fermentation or fecal elimination [205].

Overall, these mechanisms promote athletic performance by improving cardiometabolic functions, reducing recovery times and post-exercise pain, maintaining a low degree of oxidative stress, and avoiding dysregulated inflammatory processes (Table 1). Together with these canonical mechanisms, polyphenols are able, through their interaction with the intestinal microbiota, to favor the proliferation of bacterial genera of great importance for metabolic and cognitive functions such as *Akkermansia*, *Lactobacilli,* and *Bifidobacteria*. The microbiota, on the other hand, metabolizes the polyphenols in the colon to produce small bioactive molecules that exert epigenetic mechanisms on biochemical pathways by modulating gene expression (Figure 3).

In practice, polyphenols supplementation should be provided before or away from physical exercise and not immediately afterward, especially because post-exercise inflammatory processes are essential for muscle hypertrophy and learning muscle actions.

With the advent of omics technologies it has become possible to analyze the individual genome, epigenome and other classes of biologically relevant molecules, as well as the genetic composition of intestinal microbiota (microbiome). The biological data contained in the genetic/epigenetic fingerprint and the individual microbiota composition, together, provide invaluable information to understand a subject’s own sensitivity and response to external/internal stimuli and dietary xenobiotics (Figure 1). This, in turn, can allow personalized interventions in every medical field, including sports medicine, where personalized nutritional and nutraceutical regimes can be realized to maximize athletic performances.

Today, there are still few data on the role of genetic polymorphisms and microbiota influence on polyphenols absorption, distribution, metabolism and elimination, and the impact on athletic performance. In many cases, the application of nutritional genomics to sports performance is extrapolated from data of single genetic polymorphisms analyzed in reference to other conditions, or in specific diseases, so it will be necessary to confirm the relevance of these data in athletes.

Research on the effects of polyphenols on the human epigenome and their bidirectional interactions with microbiota is in its infancy, but thanks to omics techniques and the combined use of a systems biology approach, it is possible today to rapidly reach a critical mass of knowledge for their targeted use also in the field of sports medicine. Individual network analysis comparing gene-nutrient associations and genetic polymorphisms, also taking into consideration the individual microbiota and its role in xenobiotics bioavailability, will likely allow more precise and personalized strategies in athletes for effective nutritional regimens, phytocomplex supplementation/integration and targeted exercises. Within this context, that can be indicated collectively as phytonutritional epigenomics (Figure 3), polyphenols are likely candidates for safe and effective modulation of biological phenomena of interest for the improvement of sports performance.

Polyphenol glycosides are hydrolyzed by two main enzymatic mechanisms in the small intestine: Lactase phenylin hydrolase (LPH), an enzyme present on the brush border of the epithelial cells of the small intestine, and cytosolic β-glucosidase (CBG), an enzyme located within the epithelial cells where the polar glucosides are transported through the active sodium-dependent glucose transporter 1 (SGLT1). However, this path is less relevant due to the presence of an intracellular glucose outflow system directed towards the lumen of the digestive tract mediated by the transporter Multidrug Resistance Associated Protein 2 (MRP2) that limits polyphenols absorption [56,205]. Once absorbed in the small intestine, residual polyphenolic compounds undergo Phase I enteric and hepatic biotransformation (oxidation, reduction, and hydrolysis) and, subsequently, Phase II reactions (conjugation) [203,205]. These transformations generate a plethora of water-soluble conjugated metabolites (glucuronide, sulfate and methyl derivatives), rapidly released into the systemic circulation for subsequent release in the organs [206,207]. In the large intestine, microbial enzymes influence the 90–95% unabsorbed polyphenols and sequentially produce metabolites with various physiological consequences. Colon microflora transforms residual polyphenols into bioactive compounds that influence intestinal ecology and affect human health [10,208]. Legend: LPH (lactase-phlorizin hydrolase); SGLT1 (sodium-dependent glucose cotransporter 1); CBG (cytosolic beta-glucosidase); ABC (ATP-binding cassette); SCFA (short-chain fatty acids); OPC (oligomeric proanthocyanidins); EGCG (epigallocatechin-3-gallate).

## Figures and Tables

**Table 1:** Average daily dose and overall benefits in humans of polyphenol supplementation in sports performance.

	Average Daily Dose	Overall Benefits	References
Curcumin	80–200 mg	- reduces muscle fatigue, muscle mass loss, muscle soreness, and post-exercise recovery; - ameliorates redox homeostasis and insulin sensitivity	[59,65,68]
Resveratrol	100–500 mg	- improves muscle strength and fatigue tolerance, and muscle regeneration after disuse; - increases skeletal muscle mitochondrial capacity; - exerts ergogenic, and anti-obesity properties; increases fatty-acid beta-oxidation and glucose metabolism; - improves glucose control and insulin sensitivity in diabetic or prediabetic subjects without altering glycemic measures in nondiabetic individuals	[100,109,114,119]
Cocoa Flavanols	200–500 mg	- induces vasodilation, improves endothelial function and reduces blood pressure; - increases cerebral blood flow; - improves vascular function; - reduces exercise-induced oxidative stress; - alters fat and carbohydrate utilization during exercise without affecting athletic performance;	[131,132]
Quercetin	200–1000 mg	- increases athletic performance and energy expenditure; - boosts both physical and mental performance; - improves neuromuscular performance during and after resistance training sessions; - attenuates muscle weakness severity caused by eccentric-induced myofibrillar disruption and sarcolemmal action potential propagation impairment; - reduces post-stroke muscle pain, localized pain, oxidative stress, cramps, and post-exercise recovery time;	[146,148,150,153]
Green tea extract	250–1000 mg	- reduces muscle damage and oxidative stress with positive effects on neuromuscular parameters on muscle fatigue;	[156,157,158]
Blueberry	75–150 g	- improves recovery after exercise; - improves vascular functions and vasodilation;	[160,161]
Pycnogenol^®^	100–800 mg	- improves physical performance and protect from oxidative stress post-exercise; improve training and performances both in normal subjects and in semi-professional athletes performing at high levels in difficult, high-stress sports such as the triathlon.	[171]
Montmorency cherry juice	30 mL	- Increases muscle recovery, and reduce post-exercise pain mainly in strength sports;	[174,177]
Ecklonia cava polyphenols	40 mg	- increases glucose oxidation; - reduces lactate production during intense exercise;	[178]
